# Microbial-Based Therapies in the Treatment of Inflammatory Bowel Disease – An Overview of Human Studies

**DOI:** 10.3389/fphar.2018.01571

**Published:** 2019-01-10

**Authors:** Paulo José Basso, Niels Olsen Saraiva Câmara, Helioswilton Sales-Campos

**Affiliations:** ^1^Department of Immunology, Institute of Biomedical Sciences, University of São Paulo, São Paulo, Brazil; ^2^Institute of Tropical Pathology and Public Health, Federal University of Goiás, Goiânia, Brazil

**Keywords:** fecal microbiota transplantation, probiotics, Crohn’s disease, Ulcerative colitis, dysbiosis

## Abstract

Inflammatory bowel disease (IBD) is a group of multifactorial and inflammatory infirmities comprised of two main entities: Ulcerative colitis (UC) and Crohn’s disease (CD). Classic strategies to treat IBD are focused on decreasing inflammation besides inducing and extending disease remission. However, these approaches have several limitations such as low responsiveness, excessive immunosuppression, and refractoriness. Despite the multifactorial causality of IBD, immune disturbances and intestinal dysbiosis have been suggested as the central players in disease pathogenesis. Hence, therapies aiming at modulating intestinal microbial composition may represent a promising strategy in IBD control. Fecal microbiota transplantation (FMT) and probiotics have been explored as promising candidates to reestablish microbial balance in several immune-mediated diseases such as IBD. These microbial-based therapies have demonstrated the ability to reduce both the dysbiotic environment and production of inflammatory mediators, thus inducing remission, especially in UC. Despite these promising results, there is still no consensus on the relevance of such treatments in IBD as a potential clinical strategy. Thus, this review aims to critically review and describe the use of FMT and probiotics to treat patients with IBD.

## Introduction

Inflammatory Bowel Disease (IBD) is a group of immune-mediated diseases mainly represented by Ulcerative colitis (UC) and Crohn’s disease (CD) (Mao et al., 2018). IBD presents a multifactorial etiology driven by immunological disturbances, genetic alterations and the influence of environmental factors such as diet, lifestyle, socioeconomic development, intestinal dysbiotic microbiota, among other aspects (Basso et al., 2014). Current therapies are based on pharmacological approaches using traditional medicines such as aminosalicylates, corticosteroids, thiopurines, folic acid antagonists, or biological therapies, aiming at controlling inflammation besides reducing disease relapse (Sales-Campos et al., 2015). However, these approaches are not curative, and patients may become refractory or intolerant to them. In this context, therapies aiming at modulating the microbes inhabiting the human body, especially the intestine, have been suggested as one of the most promising strategies to treat immune-mediated diseases such as IBD (Ott et al., 2004; Alipour et al., 2016). This is of particular interest because recent investigations demonstrate that conventional treatments fail to completely restore the normal microbiota of patients with IBD, even if associated with special diets (Lewis et al., 2015). Though we understand the importance of other microbial interventions using symbiotics and prebiotics, for example, this review will focus on the human studies using fecal microbiota transplantation (FMT) and probiotics as strategies to restore the normal microbiota in IBD patients.

## Intestinal Microbiota

Before addressing the role of FMT and probiotics in IBD, it is important to introduce how the intestinal microbiota is able to interact with the vertebrate host, thus influencing health and disease status. Despite the great distribution of microorganisms in different sites of the human body, the most diverse microbial species is found in the gastrointestinal tract (GIT) (Hooper and Gordon, 2001; Hooper et al., 2001). More than 1000 microbial species, including bacteria, virus, and fungal, were identified in the human GIT (Turnbaugh et al., 2007). These commensal and symbiotic communities of microorganisms, also known as microbiota, are able to directly or indirectly influence local and systemic physiology of the human body, including but not limited to the immunologic, endocrine, and nervous systems (Lei et al., 2015). The composition of gut microbiota, in turn, can be influenced by different aspects such as diet, xenobiotics, lifestyle, and genetics (Goodrich et al., 2014; Wen and Duffy, 2017). Thus, it is reasonable to assume the great impact that perturbations in the complex bidirectional relationship between vertebrate hosts and gut microbes may have on host physiology. Further, this complex interaction can also lead to the onset and maintenance of several diseases, including IBD (Eck et al., 2017). Though gut microbiota is colonized by different microorganisms (bacteria, fungi, archaea, and viruses), the term “microbiota” is often used to refer to bacterial species within the GIT, which represents more than 96% of the total microbial population (Turnbaugh et al., 2007). However, fungal and viral dysbiosis have also been implicated in IBD development (Lewis et al., 2015; Duerkop et al., 2018).

To limit inappropriate activation in surfaces with great contact with microbes, like GIT, the human body has developed chemical and physical barriers to anatomically separate the microbiota from immune cells (Hooper et al., 2012). However, this interface is not insurmountable and some commensal microorganisms are able to interact with the immune, endocrine and nervous systems (Cani and Knauf, 2016). So far, two hypotheses were proposed to clarify the mechanisms concerning this interplay: the presence of pattern recognition receptors (PRR) in host cells sensing microbial associated molecular patterns (MAMPs)/danger associated molecular patterns (DAMPS), and the activity of microbial metabolites over different mammalian biological systems (Castro et al., 2015; Rangan et al., 2016). In this context, it is possible to highlight the beneficial role of the polysaccharide A of *Bacteroides fragilis*, which is able to stimulate the differentiation and activity of regulatory T cells (Treg) in the gut (Donaldson et al., 2016). The presence of Tregs in intestine is of great contribution to the maintenance of a tolerant environment, thus avoiding unnecessary inflammation (Hoeppli et al., 2018). Further, the production of immunoglobulin A (IgA) by intestinal plasma cells, which is crucial for the protection against pathobionts in intestine, is positively influenced by epithelium-associated bacteria such as *Mucispirillum* and segmented filamentous bacteria (SFB) (Bunker et al., 2015). One of the most studied groups of microbiota-derived metabolites with protective effects toward the mammalian host is the short chain fatty acids (SCFAs) that are mainly derived from fermentation of dietary fibers (Rios-Covian et al., 2016). SCFAs are primarily represented by three compounds acetate, propionate and butyrate, which contribute to the integrity of intestinal epithelium besides directly influencing host metabolic and immune functions (van de Wouw et al., 2018).

## Intestinal Dysbiosis in the Pathogenesis of IBD

Dysbiosis has been explored as a causative agent of several systemic and local diseases affecting GIT, including UC and CD (Kostic et al., 2014). The gut microbial changes in IBD are summarized in Table 1. In comparison to healthy subjects, IBD patients have reduced microbial composition (up to 25%), diversity, and richness with increased numbers of pathogenic/pathobionts microorganisms (e.g., Proteobacteria, Fusobacteria species, and *Ruminococcus gnavus –* Firmicutes) (Frank et al., 2007), and decreased numbers of beneficial microorganisms such as *Lachnospiraceae* (Firmicutes), *Bifidobacterium* species (Actinobacteria), *Roseburia* (Firmicutes), *Sutterella* (Proteobacteria) (Gilbert et al., 2016), and *Faecalibacterium prausnitzii* (Firmicutes), which are at least 10-fold reduced in IBD (Xiao et al., 2015). To note, *F. prausnitzii* has been suggested as one of the major microbial components of human healthy intestinal microbiota representing almost 5% of the total bacterial population (Louis and Flint, 2009). This bacterium contributes to the maintenance of a regulatory environment in intestine through the production of butyrate, besides providing energy to colonocytes (Sokol et al., 2008). The observation that intestinal microbes cooperate to the maintenance of epithelial integrity in intestine is of great importance since these mechanisms are frequently disrupted in IBD. One of the theories to explain the occurrence of dysbiosis in IBD relies on the inflammation. Results from both experimental and clinical investigations associate inflammatory responses and perturbations in microbial composition in ileum and other intestinal areas to the development of dysbiosis (Gevers et al., 2014; Forbes et al., 2016). On the other hand, a less dysbiotic environment is observed in non-affected areas of diseased subjects (Forbes et al., 2016).

**Table 1:** Changes in gut microbiota composition in inflammatory bowel disease patients.

Microorganism (s)	Commensal (C) or pathogenic (P) microorganisms^∗^	UC	DC
Verrucomicrobia	C	↓	↓
Bifidobacterium	C	↓	↓
Roseburia species	C	↓	?
Bacteroides	C	↓↑	↑
Firmicutes	C	↓	↓
Clostridium species (clusters IV and XIVa	C	↓	↓↑
*Saccharomyces cerevisiae*	C	↓	↓
Pseudomonas	P	↓	↓
Proteobacteria	P	↑	↑
Fusobacterium	P	↑	↑
*Ruminococcus gnavus*	P	↑	↑
*Candida albicans*	P	↑	↑

*CD, Crohn’s disease; UC, Ulcerative colitis. ^∗^Most of the species. References (Gophna et al., 2006; Frank et al., 2007; Kaakoush et al., 2012; Machiels et al., 2014; Lewis et al., 2015; Tahara et al., 2015; Shah et al., 2016; Sokol et al., 2017; Vrakas et al., 2017).*

There is evidence suggesting dysbiosis as a cause of IBD. Environmental factors, which directly affects intestinal microbiota composition, have been pointed out as one of the key players in the pathogenesis of IBD. In this regard, early life exposure to breastfeeding and maternal smoking during pregnancy, have been inversely and positively correlated to disease outcome in CD, respectively (Lindoso et al., 2018). Accordingly, patients with UC (Elinav et al., 2011) tend to have a better outcome when treated with microbial-based therapies (i.e., antibiotics, FMT, and probiotics). The mechanisms concerning the influence of dysbiosis in IBD outcome are still a matter of debate and investigation. Some studies suggest the association between the development of inflammation and the presence of some specific bacteria species. The reduction in strict anaerobes (e.g., *Clostridium* groups IV and XIVa), along with the expansion of facultative aerobic or aerobic bacteria, may increase the local concentration of oxygen, thus leading to augmented vascular and mucosal permeability, and promoting intestinal inflammation (Albenberg et al., 2014). Different strains of *Clostridium* species (e.g., IV, XIVa, and XVIII), which lack toxins and virulence factors, have their immunosuppressive activity demonstrated by inducing Treg cells in intestine in a TGF-β-, IL-10- or butyrate-dependent manner (Atarashi et al., 2013; Furusawa et al., 2013). These data suggest that microbial imbalance in IBD favors the development of inflammation by reducing crucial anti-inflammatory players, besides favoring the onset of pro-inflammatory mechanisms. On the other hand, inflammation *per se* also contributes to the onset of a dysbiotic environment. Regardless if inflammation leads to dysbiosis or vice-versa, they have a strong synergistic interaction that must be targeted to develop improved therapeutic strategies. For this reason, therapies aiming at reestablishing the microbial balance may represent the next frontier to treat inflammatory disorders, such as IBD, in which the dysbiosis plays a central role in disease pathogenesis.

## Fecal Microbiota Transplantation (FMT)

Fecal microbiota transplantation has long been used to treat recurrent *Clostridium difficile* infection (CDI) presenting great effectiveness and significant safeness, with cure rates reaching 90% (Khan et al., 2018). One of the main mechanisms proposed to explain the ability of FMT to treat CDI is attributed to its capacity to restore intestinal microbial balance (Gagliardi et al., 2018). This characteristic has expanded the use of FMT to treat both local and systemic illnesses associated with gut dysbiosis, such as irritable bowel syndrome (IBS) (Mizuno et al., 2017), IBD (Angelberger et al., 2013; Kunde et al., 2013) and metabolic syndrome (Vrieze et al., 2012).

Because of the importance of elucidating how microbiota donors are selected and how FMT is delivered to recipients, these aspects will be clarified first. Then, we are going to present and discuss the most important scientific studies regarding the therapeutic use of FMT in IBD (Table 2).

**Table 2:** Clinical trials of fecal microbiota transplantation for inflammatory bowel disease.

Authors	Diagnosis	Number of patients (P) or studies (S)^∗ #^	FMT route	Therapeutic regimen^&^	Outcome
Paramsothy et al. (2017b)	UC	*n* = 41 (S)	N.A	N.A	33% of clinical remission
	CD	*n* = 11 (S)	N.A	N.A	52% of clinical remission
Moayyedi et al. (2015)	UC	*n* = 70 (P)	Enema	50 g offeces/300 mL of water; once weekly for 6 weeks	24% of clinical remission
Paramsothy et al. (2017a)	UC	*n* = 85 (P)	Enema	150 mL^$^; once a day, 5 days per week for 8 weeks	27% of clinical and endoscopic remission or response
Rossen et al. (2015)	UC	*n* = 50 (P)	Naso-duodenal tube	60 g of feces/500 mL of saline; two doses (days 0 and 21)	No statistical difference between control and treated patients
Vaughn et al. (2016)	CD	*n* = 19 (P)	Colonoscopy	50 g of feces/250 mL of saline; one dose	58% of clinical response (control group not included)
Cui et al. (2015)	CD	*n* = 30 (P)	Endoscopy	150–200 mL^$^; one dose	86.7 and 76.7% of clinical improvement and remission, respectively at week 4
Suskind et al. (2015)	CD	*n* = 9 (P)	Nasogastric tube	30 g of feces/100 or 200 mL of saline; one dose	77.77% of clinical remission at week 2 55.55% of clinical remission at weeks 6 and 12

CD, Crohn’s disease; N.A, Not applicable; UC, Ulcerative colitis. ^*^Both total number of patients for clinical trials and number of studies for systematic analysis or meta-analysis were included. ^*#*^Includes the number of control patients. ^*&*^Feces may have undergone additional steps for FMT samples preparation. ^*$*^Initial solution concentration is not available.

### FMT Donor Screening and Routes of Administration

Several aspects must be considered in the search for microbiota donors. Prior to the gut microbial sequencing *per se*, a putative donor must be screened for the presence of infectious agents in feces, including *C. difficile*, intestinal parasites and virus (e.g., Norovirus) (Paramsothy et al., 2015). In blood, aside from the complete blood count, electrolytes, liver, and kidney function tests, the presence of inflammatory markers, and transmissible infectious agents such as HIV, Hepatitis, HTLV, among others, must be performed (Paramsothy et al., 2015). Further, as inclusion criteria, the donor must have no history of suggestive GIT disease, no other major active comorbidities, and preferably, no use of medications, especially, antimicrobials (Paramsothy et al., 2015; Holleran et al., 2018). To ensure that only healthy donors will be selected, additional criteria of exclusion must be used as follows: any family history of colorectal cancer affecting first-degree relatives; use of probiotics 3 months prior the donation period; household members with active GIT infections; any personal or familial history of malignancies, malnutrition, obesity, neurological, or developmental disorders (Paramsothy et al., 2015; Holleran et al., 2018). The difficulties to select FMT donors that fulfills all the stringency criteria along with the costs involved in the screening process have created some important barriers for the broader utilization of this microbial therapeutic approach. Unfortunately, this scenario has stimulated patients to perform FMT in a “homemade” fashion, using inappropriate screened donors, without medical supervision, which often result in serious complications (Hohmann et al., 2014).

For a long time, retention enema was the most used technique for FMT. However, alternative approaches have been used in this regard, including nasogastric tube, capsules, colonoscopy, and self-administered enemas, as previously reviewed (Allegretti et al., 2017). Colonoscopy and retention enema are by far, the most frequently used routes of FMT administration (Gough et al., 2011).

### FMT in IBD

Ulcerative colitis and Crohn’s disease are the major entities represented by IBD. The role of FMT has been more explored in the former. From the 307 adult patients pooled in a meta-analysis from 24 UC cohort studies, FMT induced remission in 33%. In 6 pediatric cohort studies, totalizing 34 UC patients, clinical remission was slightly reduced to 23% (Paramsothy et al., 2017b). Three randomized controlled trials also presented promising results regarding the use of FMT to treat UC. From a total of 70 UC patients with active disease without infectious diarrhea enrolled in the study, 36 were treated with FMT, and 34 with placebo, once a week for a total of 6 weeks, and remission was induced in 24% of those treated with FMT compared to 5% in the placebo group (Moayyedi et al., 2015). It is important to mention that both placebo and FMT groups were under concomitant anti-inflammatory/immunosuppressive therapy (e.g., corticosteroids, mesalamine, and anti-TNF therapy) while enrolled in the study (Moayyedi et al., 2015). Similar results were observed using enemas 5 days per week for 8 weeks, in a study in Australia that observed a remission rate of 27% in UC patients with active UC treated with FMT when compared to 8% in patients treated with placebo only (Paramsothy et al., 2017a). Regardless if patients had received FMT or not, they were also treated with immunosuppressive drugs such as 5-aminosalicylates, thiopurines, methotrexate, and/or oral prednisone, in a stable dose (Paramsothy et al., 2017a). On the other hand, the remission rates observed in UC patients treated with FMT from healthy donors were similar to those observed in UC patients receiving their own fecal microbiota (Rossen et al., 2015).

Unfortunately, data supporting the role of FMT in CD are scarcer than in UC, and so far, no results from randomized clinical trials are available. The evidence of the beneficial effects of FMT in CD are all derived from small and uncontrolled studies. A single dose of FMT performed by colonoscopy showed an improvement in clinical outcome of 58% of patients treated with FMT (Vaughn et al., 2016). This observation was followed by increased levels of Tregs in recipients’ lamina propria followed by higher microbial diversity (Vaughn et al., 2016), which suggests a reestablishment of microbial balance and a less prominent inflammation. Similarly, a single treatment with FMT induced clinical improvement and remission based on clinical activity in CD patients (Cui et al., 2015). This amelioration was followed by increased patient’s body weight after FMT (Cui et al., 2015). For all CD-patients enrolled in the study a 12-week washout period was required for those exposed to immunosuppressive therapies such as cyclosporine, tacrolimus, or infliximab. Antibiotics and probiotics were withdrawn 60 and 30 days before FMT, respectively (Vaughn et al., 2016). The beneficial role of FMT was also addressed in young patients with CD. Nine individuals, aged 12–19 years, presenting mild-to-moderate symptoms received FMT by nasogastric tube once and were followed by 12 weeks (Suskind et al., 2015). Based on the clinical score, 2 weeks after FMT, 7 of 9 patients were in remission, and 5 of 9 patients were in remission at 6 and 12 weeks after FMT. All patients enrolled in the study were allowed to receive immunomodulators during the FMT or placebo treatment (Suskind et al., 2015).

### Limitations in FMT Studies

The studies presented here showed promising results regarding the use of FMT to induce remission in UC and to a less extent in CD patients. The differences in the route and interval of administration, besides of the composition and bacterial load in FMT, may explain the dissimilarities observed among studies. Another important drawback is the lack of comprehensive guidelines to be used globally in the screening and standardization of putative microbiota donors (age, gender, and health status) along with strategies of production, dosage regimen and to evaluate the transplant engraftment. Further, probably because of economic reasons, clinical trials do not deeply investigate the microbial composition of fecal donors using 16S rRNA sequencing and their similarities to the recipients’ microbiota. Thus, the observation of similarities between the intestinal microbiota composition of donors and recipients may dictate the successfulness of FMT engraftment. Without the proper identification of the microbial community and the total bacterial load transplanted from a healthy donor to a diseased subject, it is difficult to predict the impact of FMT in IBD or other disorders. Further, as the majority of clinical trials were conducted with concomitant use of immunomodulatory drugs, it is reasonable to assume that FMT may work better as an adjuvant therapy rather than an isolated strategy. To confirm the role of FMT in IBD, more controlled clinical trials with a great number of patients and more standardized fecal samples must be conducted. Additionally, strategies aiming at providing an intestinal microbiota rebalance using well-defined microbial species may represent an improved alternative to total FMT.

### FMT Adverse Effects

In general, up to 10% of FMT recipients present minor to mild self-limited adverse effects. The majority of them are related to disturbances in GIT such as diarrhea and abdominal discomfort/pain (Hohmann et al., 2014; Baxter and Colville, 2016). Though less frequently observed, severe side effects can include IBD flares, CDI and other infections, colectomy, small bowel obstruction, pancreatitis, and even death, as recently reviewed (Qazi et al., 2017; Jeon et al., 2018). However, some evidences have shown no differences between FMT and control groups concerning the occurrence of undesirable effects (Narula et al., 2017). Despite the possibility of occurrence of adverse effects, FMT is considered to be safe in IBD. An in-depth screening of donors along with a broader comprehension of the physiopathology in IBD may facilitate the development of strategies to avoid the occurrence of such undesirable effects.

## Probiotics

Probiotics are used as safe food additives, pharmaceutical formulations or nutritional supplements defined as “live microorganisms which, when administered in adequate amounts, confer a health benefit on the host” by the World Health Organization (WHO) (Hill et al., 2014). Nevertheless, studies have pointed out that dead microorganisms or their biologically active compounds *per se* can also play protective functions, inferring that the “probiotic” definition should be revisited or other classifications implemented (Rachmilewitz et al., 2004).

The underlying mechanisms of probiotics are dependent on microbial strain. Moreover, the effects of probiotic mixtures may be complementary (also referred to additive) or synergistic (Ruiz et al., 2009). In general, probiotic strains produce growth factors that strengthen the gut epithelium and antimicrobial substances (e.g., SCFAs, bacteriocins, hydroperoxides, bile acids, and lactic acids) that kill harmful microorganisms (Konieczna et al., 2012a). As a consequence, cellular components (e.g., cellular wall, DNA) are released in the gut environment, which activate immune responses by enhancing the pro-inflammatory cytokines production and immunoglobulin synthesis, besides of improving macrophage and lymphocytes activity (Markowiak and Slizewska, 2017). In this regard, the use of *Bifidobacterium infantis* 35624 in human volunteers increased the amount of IL-10 and FoxP3^+^ cells (Treg) in the circulation (Haskard et al., 2001; Konieczna et al., 2012b). Although immune tolerance is a putative consequence of these enhancements, there is still no consensus on this matter (Castellazzi et al., 2013).

Non-immunological benefits associated to probiotics include the digestion and absorption processes, competition with potential pathogens for nutrients and intestinal adhesion sites, pH alterations, agglutination of pathogenic microorganisms, and sequestration of metabolic toxins (Gagliardi et al., 2018). Animal models and *in vitro* assays describe that probiotics also decrease the apoptosis, increase the mucus synthesis, tissue repair, redistribution and production of tight junctions in gut epithelial cells, thus reducing the intestinal permeability and enhancing the barrier protection and function (Caballero-Franco et al., 2007; Zyrek et al., 2007).

Lactobacillus (e.g., *reuteri, rhamnosus, casei, acidophilus, plantarum, gasseri, paracasei, johnsonii, ghallinarum*, and *crispatus*) and Bifidobacterium (e.g., *bifidum, infantis, longum, animalis, breve, lactis*, and *adolescentis*) are the most used strains in probiotics formulations, but multispecies approach has been increasingly applied (Holzapfel et al., 2001). Others strains commonly used include *Streptococcus* spp., *Lactococcus* spp., *Enterococcus* spp., non-pathogenic *Escherichia coli* (strain Nissle), and *Clostridium* ssp (Kechagia et al., 2013).

New bacteria genera and species have also been investigated showing good perspectives in preclinical trials. These bacteria are described as new-generation probiotics bringing more complexity to common probiotics in attempt to simulate FMT treatments. The new-generation probiotics comprise *Clostridium* clusters *IV, XIVa*, and *XVIII, F. prausnitzii, Akkermansia muciniphila, Bacteroides uniformis, B. fragilis*, and *Eubacterium hallii* (El Hage et al., 2017). Technological limitations are current challenges for using these bacteria as probiotics. Importantly, *Clostridium* clusters XIVa and IV are described as promoters of Treg differentiation, critical for immune tolerance as described earlier (Atarashi et al., 2011). Indeed, these bacteria are decreased in the gut of IBD patients (Sokol et al., 2006; Kang et al., 2010; Machiels et al., 2014). Although the number of Tregs is increased in the gut of IBD patients, the expansion is not sufficient to restrain the inflammatory development.

Since gut microbiota is not composed only by bacteria, some formulations and studies use yeasts as probiotics in association with bacteria strains, or even in single-drug formulations. In this context, *Saccharomyces boulardii* is the most commonly used yeast strain and has several anti-inflammatory properties (Pothoulakis, 2009).

### Criteria for New Probiotic Development

As pharmaceutical or nutraceutical products, probiotics must meet some criteria to be commercially available. Beyond efficacy, the safety properties of a given drug are the main concern of scientists and regulatory agencies (Doron and Snydman, 2015). Bacterial and yeast strains or their derived-products have distinct levels of regulations according to their purposes and must meet the requirements outlined in published and frequently updated guidelines designed by regulatory agencies (Doron and Snydman, 2015). They can be considered as food (food ingredient, medical food, and dietary supplement), drug (new drugs) or biological product (Degnan, 2008).

As in FMT, safety is a priority, since some inflammatory conditions or patients under immunosuppressive therapy increase the susceptibility to infectious complications, including sepsis (Farina et al., 2001; Riquelme et al., 2003). Probiotics must have human origin, scientifically proven positive effects, be safe even in high-risk populations, cannot cause allergies and must present good technology properties (e.g., feasible culture and large-scale production) (Markowiak and Slizewska, 2017). Several *in vitro* assays may be employed at the first glance to evaluate probiotic potential, epithelium adherence, microbicide activity, ability in reducing the number of pathogenic bacteria and resistance to antibiotic use, bile salts, stomach acids, digestive enzymes and pH (Saarela et al., 2000).

Although not mandatory, studies should also evaluate the adverse effects and drug interactions of probiotics since they have been used as adjuvant therapy in various diseases (Thomsen et al., 2018). For instance, the probiotic *E. coli* strain Nissle 1917 influences the pharmacokinetics of concomitantly taken antiarrhythmic drug amiodarone by increasing the drug bioavailability (Matuskova et al., 2014). Therefore, their presumed safety should be avoided, and the potential risks not neglected.

### Probiotics in IBD

In general, probiotics have been effectively used in treating IBD to prevent dysbiosis in patients undergoing prolonged antibiotic or immunosuppressive therapies (Zuo and Ng, 2018). Further, these microorganisms have been used as adjuvant therapy on the attempt to reverse the dysbiotic environment associated with IBD onset and worsening (Yoshimatsu et al., 2015; Tamaki et al., 2016). Although the number of clinical and experimental studies using probiotics in IBD is substantially high, lack of standard practices in therapeutic regimens, low number of samples and poor disease characterization, have limited the relevant conclusions about the efficacy of probiotics in this scenario.

Probiotics have been described as an alternative to induce and maintain the remission in UC, while low or no effects are observed in CD. The adjuvant use of multispecies probiotic VSL#3, which contains four strains of Lactobacillus (*L. casei, L. plantarum, L. acidophilus* and *L. delbrueckii* subsp. *Bulgaricus*), three of Bifidobacterium (*B. longum, B. breve*, and *B. infantis*), and one of Streptococcus (*S. salivarius* subsp. *Thermophilus*), improved the clinical symptoms in patients with mild to moderately active UC after receiving the daily dose of 3.6 × 10^12^ CFU (Tursi et al., 2010; Mardini and Grigorian, 2014). Corroborating results were observed after treating mild-to-moderate UC patients with VSL#3 alone, twice a day at the same dose described earlier (Sood et al., 2009). The maintenance of remission rates in UC was also similar in patients under single drug treatment of either non-pathogenic *E. coli* Nissle 1917 (5–50 × 10^9^/day) or mesalazine (1500 mg/day) (Kruis et al., 2004).

However, the systematic review using rigorous statistical methods showed that the beneficial effects of both VSL#3 or *E. coli* Nissle on UC are weak or inconclusive, while there is no positive association in CD (Jonkers et al., 2012), confirming the need for further new randomized controlled trials to increase the significance level of these findings.

The use of Bifidobacterium-fermented milk (containing *B. breve, B. bifidum*, and *L. acidophilus*) as adjuvant therapy to treat 20 patients (including placebo control) with mild to moderately active UC, showed significant improvement in both clinical and endoscopic activity indexes after 12 weeks (10 billion bacteria/day) (Kato et al., 2004). Interestingly, the SCFAs concentration in feces was higher in the probiotic-treated group compared to the placebo group. However, a recent study using a similar therapeutic strategy (*B. breve*- and *L. acidophilus*-containing fermented milk) showed no efficacy to treat or maintain the remission of UC in 195 patients (Matsuoka et al., 2018). In fact, the use of *B. bifidum* as single strain-containing probiotic was sufficient to increase the levels of fecal SCFAs in healthy volunteers (Gargari et al., 2016), however, the protective role in UC or CD remains unknown. Despite some discrepancies regarding the number of patients used in the studies mentioned above, the first was the only one to confirm the increased number of Bifidobacteria in the feces of probiotics-treated patients and to perform endoscopic analysis.

The treatment with *Lactobacillus* GG (18 × 10^9^ viable bacteria/day) alone or associated with mesalazine, prolonged the relapse-free period in UC patients compared to the group treated with immunosuppressant drug alone in a 12-month treatment regimen (Zocco et al., 2006). Similarly, a systematic review of randomized clinical trials showed the use of different lactic acid bacteria and Bifidobacteria as adjuvant therapy improved the course of disease and maintenance of clinical remission in UC (Saez-Lara et al., 2015).

As stated above, probiotics have poor or no effects on CD. However, studies have yielded positive results to induce remission by associating probiotics and prebiotics (defined as symbiotics) (Fujimori et al., 2007; Saez-Lara et al., 2015). Additionally, one open-label pilot study containing four children with mildly to moderately active CD had a significant improvement on clinical aspects after treatment with *Lactobacillus* GG (10^10^ CFU/tablet, twice a day for 6 months) (Gupta et al., 2000). However, the low number of samples and the absence of appropriate control (placebo-treated patients or under regular therapy) undermine the rigor of study. Probiotics have no effects in maintaining the remission of CD (Bousvaros et al., 2005; Bourreille et al., 2013).

In conclusion, probiotics are potential options in inducing and maintaining remission of mild to moderately UC, however, seem to be ineffective in DC (Table 3). The results must be considered as preliminary evidence and further randomized double-blind placebo-controlled multicenter trials must be performed to increase the reliability of results.

**Table 3:** Effective clinical trials using probiotics for treating inflammatory bowel disease.

Authors	Diagnosis	Number of patients (P) or studies (S)^∗ #^	Probiotic	Therapeutic regimen	Outcome
Mardini and Grigorian, 2014	UC	*n* = 5 (S) *n* = 441 (P)	VSL#3^&^	Oral; 3.6 × 10^12^ CFU/day^$^	53.4% of clinical responsiveness and 43.8% of clinical remission
Tursi et al., 2010	UC	*n* = 144 (P)	VSL#3^&^	Oral; 3.6 × 10^12^ CFU/day; once a day for 8 weeks	53.4% of clinical improvement and 47.3% of clinical remission
Sood et al., 2009	UC	*n* = 147 (P)	VSL#3^&^	Oral; 3.6 × 10^12^ CFU/dose; twice a day for 12 weeks	51.9% of clinical improvement and 42.9% of clinical remission at 12 weeks
Kruis et al., 2004	UC	*n* = 327 (P)	*Escheria coli* Nissle 1917	Oral; 5–50 × 10^9^ viables bacteria; once a day for 12 months	No differences between probiotic- and mesalazine-treated groups
Kato et al., 2004	UC	*n* = 20 (P)	Fermented milk (*B. breve, B. bifidum* and *L. acidophilus*)	Oral; 10^9^ bacteria/day; once a day for 12 weeks	70% of clinical responsiveness and 40% of clinical remission
Zocco et al., 2006	UC	*n* = 187 (P)	*Lactobacillus* GG	Oral; 9 × 10^9^ viable bacteria/dose; twice a day for 12 months	No differences between probiotic- and mesalazine-treated groups
Fujimori et al., 2007	CD	*n* = 10 (P)	*B. breve, L. asei* and *B. longum*	Oral; 75 × 10^9^ bacteria/day; once a day for 13 (±4.5) months	70% of clinical responsiveness and 60% of clinical remission
Gupta et al., 2000	CD	*n* = 4 (P)	*Lactobacillus* GG	Oral; 10^10^ CFU/dose; twice a day for 6 months	75% of clinical improvement at weeks 4 and 12

CD, Crohn’s disease; UC: Ulcerative colitis. ^*^Both total number of patients for clinical trials and number of studies for systematic analysis or meta-analysis were included. ^*#*^Includes the number of control patients. ^*&*^VSL#3 is composed by *L. casei, L. plantarum, L. acidophilus, L. delbrueckii* subsp. *bulgaricus, B. longum, B. breve, B. infantis* and *Streptococcus sulivarius* subsp. *thermophiles.*^*$*^Length of treatments not available.

### Limitations on Probiotics Studies

Different therapeutic regimen (including dose and frequency of administration) is an important problem to design treatment protocols. Although doses vary according to bacterial strains, studies have shown that 10^8^–10^10^ CFU/day are ingested after consuming 100 mL or 100 g of probiotic-containing product (Atarashi et al., 2011). As a consequence, meta-analysis studies have several biases to compare related clinical trials and to draw relevant conclusions.

Unlike FMT therapy, the route of administration is not a potential problem, since the majority of studies use the oral route as the main one, although enemas are also a potential method of probiotic delivery (Oliva et al., 2012).

Another important issue regarding probiotics formulations is the quality control. Several inconsistent data have been described between label information and product content, contamination, poor quality of strains, among others, as previously reviewed (Kolacek et al., 2017). Moreover, the same strain may show different efficacy in distinct batches as a result of a lack of standardization in bacterial culture procedures used throughout the studies and manufactures. Thus, both guidelines and improvements on supervision are highly encouraged to provide sufficient information on the design of new studies and to prevent unwanted and conflicting outcomes.

The immunosuppressive therapy is also a current challenge for clinicians and researchers. Since long-term use of immunosuppressants causes dysbiosis, it is important to determine whether this factor is a premise for the patient’s responsiveness to probiotic treatment (Bhat et al., 2017).

Altogether, these factors represent important limitations in studies setup and the conflicting clinical results found in the literature may derive from poorly designed and standardized studies.

## Conclusion and Further Directions

Both FMT and probiotics are therapies with good prospects in the medical field, especially in IBD. However, like other newly developed therapies, the challenges encountered for increasing the reliability, safety, and standardization of FMT and probiotics are considerable. Thus, more multicenter studies must be performed to increase the number of samples and variables (features of IBD, phenotypic and genotypic characteristics of the patients, standardizations in the therapeutic regimen, etc.), generating more significant conclusions.

## Author Contributions

PB wrote the manuscript, edited, generated the tables, and performed the literature review. NC contributed to review the manuscript and approval of the final version. HS-C designed the aim of the review, wrote the manuscript, performed the literature review, edited and contributed to final approval for the version to be published.

## Conflict of Interest Statement

The authors declare that the research was conducted in the absence of any commercial or financial relationships that could be construed as a potential conflict of interest.
